# A Tribute to Bernardo Ochoa, MD

**DOI:** 10.3389/fped.2017.00093

**Published:** 2017-04-27

**Authors:** Ricardo González

**Affiliations:** ^1^Pediatric Surgery and Urology, Auf der Bult Kinder- und Jugendkrankenhaus, Hannover, Germany

**Keywords:** Bernardo Ochoa, urofacial syndrome, Sociedad iberoamericana de Urología Pediátrica, urología pediátrica

The sadness related to the recent death of Dr. Bernardo Ochoa prompted me to write this note regarding my interaction with this remarkable man.

Born in Barbosa, Antioquia, Colombia on September 14, 1926, he studied Medicine at the Universidad de Antioquia and graduated in 1954. He trained in general surgery at the Hospital Universitario San Vicente de Paúl and soon became interested in pediatric surgery, a field then in its infancy.

He then went on to Boston in 1959 and spent 2 years in the service of Dr. Robert Gross at Boston Children’s Hospital. The chief resident at the time was one of my mentors, Dr. W. Hardy Hendren. Upon his return to Medellín in 1960, he founded the first Department of Pediatric Surgery in Colombia. He was also Dean of the medical school of the Universidad de Antioquia in 1975–1977. He retired in 1996.

My association with Dr. Ochoa started in the mid-80s shortly after my promotion to professor, while I was becoming established as a pediatric urologist at the University of Minnesota. I remember the day my secretary announced the visit of a gentleman from Colombia. A distinguished looking man in his late 50s or early 60s came into my office who told me he was a pediatric surgeon from Medellin, was visiting his son for some weeks, and wished to observe some clinical activities.

It was not long thereafter that he told me he wished to show me some slides of patients he had treated in Colombia. The pictures of children and young adults with strange facial expressions when they laughed (Figure 1) who had developed renal insufficiency because of bladder dysfunction greatly impressed me. Some patients were incontinent, some had undergone urinary diversion, and perhaps one had died.

**Figure 1:**
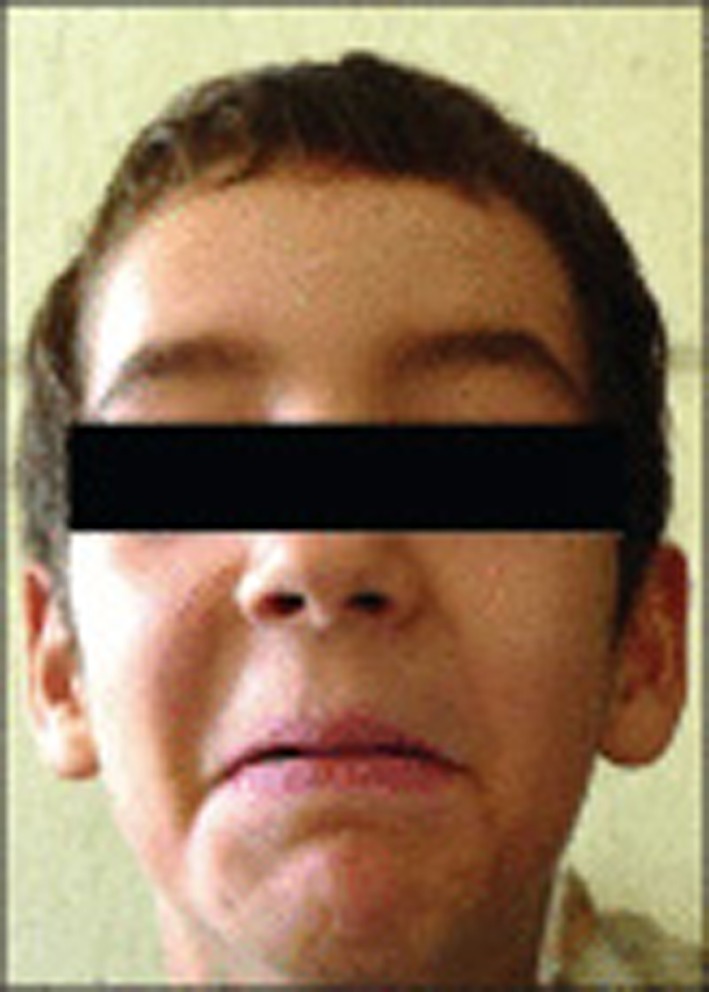
**Photograph of a child who while smiling shows a facial expression that resembles crying**. From Stamatiou and Karakos (1).

I shared this information with the chairman of the Department of Urology, Dr. Fraley, who thought it was a remarkable finding and suggested that Dr. Ochoa should share the information with Dr. Robert Gorlin (Figure 2).

**Figure 2:**
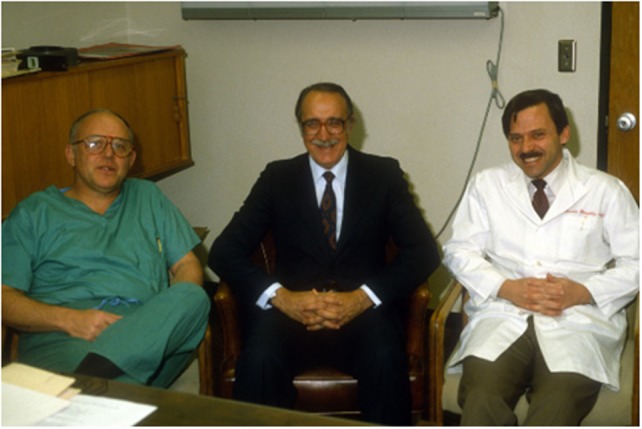
**From left to right: Dr. Elwin E. Fraley, Dr. Bernardo Ochoa, and Dr. Ricardo González circa 1982, during Dr. Ochoa’s visit to the Department of Urology at the University of Minnesota**.

Dr. Gorlin (1923–2006) was then professor of dental pathology and was well known for his expertise in craniofacial manifestation of syndromes. By then, he had authored some books on the subject and was a highly respected member of the faculty.

The result of this encounter resulted in the publication in the American Journal of Medical Genetics of the article entitled urofacial (ochoa) syndrome by Ochoa and Gorlin (2).

Although the syndrome had been published 8 years earlier by Elejade, giving credit to Ochoa in the title of the article (3), it had not received widespread recognition nor was Ochoa, who had made the observation, an author of the paper.

The significance of Ochoa and Gorlin paper is often underestimated. It is not merely a description of a rare condition. On the contrary, the urologic problems in the Ochoa syndrome are identical to those in the severe forms of the so called “non-neurogenic-neurogenic bladder” also known as the Hinman syndrome. This syndrome was long thought to be the results of behavioral problems triggered by psychological trauma. This belief led to ineffective behavioral modification managements and the waste of precious time before instituting treatments to prevent renal damage such as intermittent catheterization.

The eventual recognition is that the Ochoa syndrome had both a genetic and neurological basis meant to me that a neurologic defect in the “non-neurogenic-neurogenic bladder” had to be present although it was unrecognized with the available diagnostic methods. Thus, the treatment should not be behavioral but indeed similar to the treatments we use for neurogenic bladder of other etiologies, the effectiveness of which has been proven beyond question.

My interaction with Bernardo also led also to discussions related to the state of pediatric urology in Latin America. By then, the society that represented the specialty in this part of the world, Sociedad Latino Americana de Urología Infantil (SLAUI), excluded pediatric surgeons. I had unsuccessfully tried to change this situation by incorporating pediatric surgeons to the society but met with strong resistance from the urological community. My discussion with Bernardo encouraged me to organize a meeting in Panama in 1991, the First Inter-American Meeting on Pediatric Urology that brought together urologists and pediatric surgeons with interest in pediatric urology from the Spanish speaking world. This meeting was followed in 1993 by the Second Inter-American Meeting on Pediatric Urology in Viña del Mar, Chile that was the seed for the later foundation of the Sociedad Iberoamericana de Urología Pediátrica (SIUP) and the eventual demise of the SALUI. The SIUP is now the society which represents Spanish speaking pediatric urologists. Bernardo Ochoa was its first president (1994–1995).

The name of Dr. Ochoa will be forever engraved in the history of Latin American Pediatric Surgery and Urology and indeed in the history of Pediatric urology and nephrology in the world.

## Author Contributions

The author confirms being the sole contributor of this work and approved it for publication.

## Conflict of Interest Statement

The author declares that the research was conducted in the absence of any commercial or financial relationships that could be construed as a potential conflict of interest.
